# Rare Sugars: Recent Advances and Their Potential Role in Sustainable Crop Protection

**DOI:** 10.3390/molecules26061720

**Published:** 2021-03-19

**Authors:** Nikola Mijailovic, Andrea Nesler, Michele Perazzolli, Essaid Aït Barka, Aziz Aziz

**Affiliations:** 1 Induced Resistance and Plant Bioprotection, USC RIBP 1488, University of Reims, UFR Sciences, CEDEX 02, 51687 Reims, France; nikola.mijailovic@univ-reims.fr (N.M.); ea.barka@univ-reims.fr (E.A.B.); 2 Bi-PA nv, Londerzee l1840, Belgium; andrea.nesler@bi-pa.com; 3 Department of Sustainable Agro-Ecosystems and Bioresources, Research and Innovation Centre, Fondazione Edmund Mach, 38010 San Michele all’Adige, Italy; michele.perazzolli@unitn.it; 4 Center Agriculture Food Environment (C3A), University of Trento, 38098 San Michele all’Adige, Italy

**Keywords:** rare sugars, crop protection, resistance inducers, plant immunity, biobased pesticides, sustainable agriculture

## Abstract

Rare sugars are monosaccharides with a limited availability in the nature and almost unknown biological functions. The use of industrial enzymatic and microbial processes greatly reduced their production costs, making research on these molecules more accessible. Since then, the number of studies on their medical/clinical applications grew and rare sugars emerged as potential candidates to replace conventional sugars in human nutrition thanks to their beneficial health effects. More recently, the potential use of rare sugars in agriculture was also highlighted. However, overviews and critical evaluations on this topic are missing. This review aims to provide the current knowledge about the effects of rare sugars on the organisms of the farming ecosystem, with an emphasis on their mode of action and practical use as an innovative tool for sustainable agriculture. Some rare sugars can impact the plant growth and immune responses by affecting metabolic homeostasis and the hormonal signaling pathways. These properties could be used for the development of new herbicides, plant growth regulators and resistance inducers. Other rare sugars also showed antinutritional properties on some phytopathogens and biocidal activity against some plant pests, highlighting their promising potential for the development of new sustainable pesticides. Their low risk for human health also makes them safe and ecofriendly alternatives to agrochemicals.

## 1. Introduction

Monosaccharides can be found in a large variety of stereoisomer forms in the nature. Isomers such as D-glucose and D-fructose, the classical examples of natural sugars, are exceptions as they exist in great abundance. However, the vast majority of their stereoisomers are hard to isolate from natural sources or to synthesize chemically owning to their complex structures [1,2]. According to the definition of the International Society of Rare Sugars (ISRS), such carbohydrates represent a group of different monosaccharides and their derivatives that are found in low abundance in nature and they are called rare sugars [3,4,5]. A list of 42 known monosaccharides has been compiled [5]. Some of them can be produced while others are already present in the nature. The list comprises 24 hexoses, 12 pentoses and 6 tetroses, half of them with D-configuration and half with L-configuration. Only 7 out of these 42 monosaccharides are considered as nonrare sugars, such as D-glucose, D-fructose, D-galactose, D-mannose, D-ribose, D-xylose and L-arabinose [6]. Thus, twenty hexoses (including D-allulose, D-allose, D-sorbose and D-tagatose) and nine pentoses (including D-lyxose, L-xylulose, and D-xylitol) have been classified as rare sugars by the ISRS [7,8]. Moreover, disaccharides can be produced from the listed sugars, which may differ depending on the type of monosaccharide and the position of the glycosidic bond. Examples of rare disaccharides are turanose, leucrose, isomaltulose, kojibiose, nigerose, isomaltose, sophorose, laminaribiose and gentiobiose [9]. Due to the low quantity and availability in nature, knowledge about the ecological role and effect of rare sugars on living organisms remained scarce [10,11]. This lack raises the question of whether rare sugars have a biological function [9]. For example, D-trehalose is a nonreducing disaccharide known to serve as the main sugar component of hemolymph in insects [12] and as a potential signal metabolite in yeast and plants during exposure to biotic and abiotic stresses [13,14,15,16].

**Figure 1:**
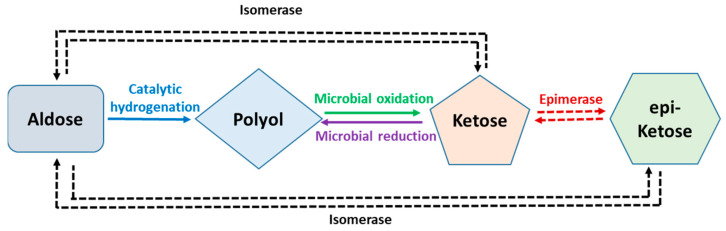
Strategy for interconversion of all the monosaccharides developed by Izumori group; modified from Best et al. [17].

One of the major obstacles limiting the use of rare sugars, in addition to their low availability, is the limited and quite expensive synthesis methods. Izumori and colleagues developed a methodology for the cost- and time-effective production of rare sugars, called Izumoring (Figure 1) [3]. This includes the production process of ketohexoses, aldohexoses and hexitols using enzymatic and microbiological reactions [3,4]. In short, isomerases are used to equilibrate aldoses with their corresponding keto forms, and aldoses are reduced to their corresponding polyols by catalytic hydrogenation. Microbial oxidation is used to transform polyols into single ketoses and the enzymatic epimerization of ketoses yields epimeric ketoses [17]. The Izumoring strategy combines the microbial oxidation of polyols to their corresponding ketoses, which is followed by the subsequent epimerization by D-tagatose-3-epimerase [18,19,20]. D-tagatose-3-epimerase is used to interconvert any ketohexoses that are epimeric at carbon-3 location [4,5]. Polyol dehydrogenase is then used to catalyze oxidation–reduction reactions between ketohexoses and the corresponding hexitols [21]. Furthermore, the stereochemical arrangement plays a role because oxidases derived from different microorganisms have different configurational requirements for oxidation.

**Figure 2:**
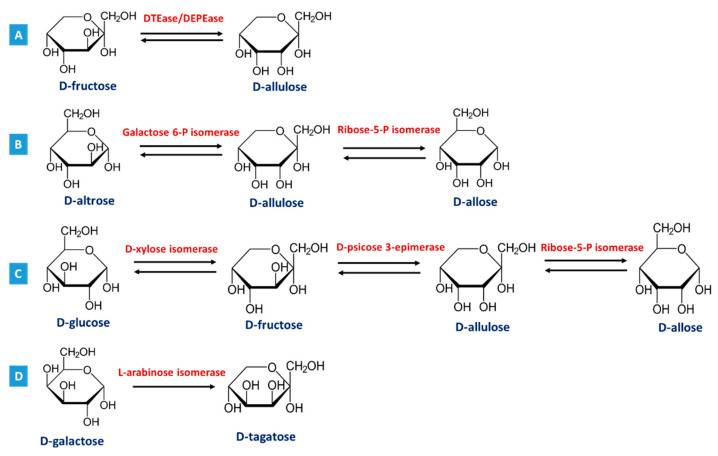
Enzymatic bioconversion of (**A**) D-fructose to D-allulose catalyzed by D-tagatose/D-psicose 3-epimerase (DTE/DPEase) enzymes; (**B**) D-allulose to D-allose, with or without D-arabinose as a byproduct when catalyzed by Galactose 6-phosphate isomerase or Ribose-5-phosphate isomerase, respectively; (**C**) D-glucose to D-allose sequentially catalyzed by D-xylose isomerase, D-psicose 3-epimerase, and ribose-5-phosphate isomerase; and (**D**) D-galactose to D-tagatose catalyzed by L-arabinose isomerase enzymes, according to Li et al. [11].

In the Izumoring system, four different microorganisms (*Gluconobacter thailandicus*, *Enterobacter aerogenes*, *Klebsiella pneumoniae* and *Enterobacter agglomerans*) are used [22,23,24], whereby all ketohexoses, aldohexoses and hexitols are linked to make a symmetric ring structure, which allows the production of 34 hexoses. For example, D-allulose can be prepared through the epimerization of D-fructose at C-3 which is catalyzed by D-tagatose 3-epimerase family enzymes (DTEase, EC 5.1.3) [11] (Figure 2A), derived from *Pseudomonas* sp. ST-24 [18], *Rhodobacter sphaeroides* SK011 [25], *Clostridium cellulolyticum* H10 and *Clostridium scindens* 35704 [26,27], but also, D-psicose 3-epimerase (DPEase, EC 5.1.3) derived from *Agrobacterium tumefaciens* [28]. D-allose can be produced through enzymatic bioconversion of D-allulose [11], catalyzed by L-rhamnose isomerase (EC 5.3.1.14) from *Pseudomonas stutzeri* [29] and galactose 6-phosphate isomerase (EC 5.3.1.26) from *Lactococcus lactis* [30] with D-altrose as a byproduct, or catalyzed by L-rhamnose isomerase (EC 5.3.1.14) from *Bacillus pallidus* [31] and ribose-5-phosphate isomerase (EC 5.3.1.6) from *Clostridium thermocellum* [32] without having D-altrose as a byproduct (Figure 2B). The D-glucose (which is much cheaper than D-allulose) can also be used as a starting material to synthesize D-allose through a three-step bioconversion, catalyzed by D-xylose isomerase (EC 5.3.1.5), D-psicose 3-epimerase, and ribose-5-phosphate isomerase (Figure 2C) [11]. D-tagatose can be produced through bioconversion from D-galactose catalyzed by L-arabinose isomerase enzymes (AI, EC 5.3.1.4) (Figure 2D) [11] derived from *Geobacillus stearothermophilus* [33], *Thermotoga neapolitana* [34] or *Saccharomyces cerevisiae* [35].

## 2. Rare Sugars in Food Systems and Medicine

There is growing evidence demonstrating that rare sugars are already very successful in the food market and in human health, attracting the attention of many research and commercial organizations [36]. Some rare sugars, such as D-allulose, D-allose and D-tagatose, have found their route to industrialization and commercialization in the food industry as low calorie sweeteners [36]. D-allulose (previously called D-psicose) is a bioactive epimer of D-fructose at the C-3 position. D-allulose is found in wheat [37], processed cane and beet molasses [38], steam-treated coffee [39] and heated fruit juice [40]. D-allulose is also present in certain bacteria [25] but so far not in animals [41]. D-allulose has been approved as “Generally Recognized as Safe” (GRAS), allowing its usage in a range of food products and dietary supplements [40]. Due to its unique physicochemical properties, D-allulose is an excellent alternative to D-sucrose in foods [40]. It is approximately 30% less sweet compared to sucrose [36,40], but it contains almost no calories (it has only 0.3% of the energy of sucrose) [42]. In comparison with D-fructose and D-glucose, D-allulose has a much stronger antioxidative activity that persists over a long period of storage [43,44,45,46], and is highly soluble [47]. D-allulose showed hypoglycemic properties and therapeutic effects on type 2 diabetes [48,49,50,51,52,53,54,55,56,57]. It also has antiobesity [54,55,58,59] and antihyperlipidemic effects [42,60]. Furthermore, D-allulose can be used against inflammation [61] and atherosclerotic diseases [62], as a neuroprotectant [63] and assist in pulmonary drug delivery [64]. Interestingly, D-allulose is also the foremost identified sugar with anthelmintic properties that effectively suppresses the growth of parasites, such as trichomonads [65]. When combined with metronidazole, D-allulose greatly improved its efficacy against trichomonad parasites [65,66]. In animals, long-term administration of D-allulose did not cause adverse and toxic effects on hematological and chemical parameters [59]. Likewise, it has been found that D-allulose did not cause any abnormal effects or clinical problems in humans over long period of continuous ingestion [67,68].

The other rare sugar of great interest is D-allose, a C-3 epimer of D-glucose found in various plant species, such as *Protea rubropilosa* [69,70], *Veronica filiformis* [71], *Mentzelia* spp. [72], *Solanum tuberosum* [73], *Halodule pinifolia* [74], *Acalypha hispida* [75], *Tamarindus indica* and *Crataeva nurvala* [76]. D-Allose is a low-calorie sweetener, with 20% less sweetness than sucrose, which can be easily dissolved in water [59,77]. Cancer and tumor inhibition is considered to be the most important property of D-allose [78]. D-allose has been reported to be effective against various human cancers, such as ovarian [79], cervical and skin [80], hepatocellular [81,82] and prostate [83,84] cancers. Leukemia [10], head and neck cancer [85], pancreas [86], and lung cancer [87] are also inhibited by D-allose. Furthermore, radiation, when coupled with D-allose, stimulates the production of reactive oxygen species (ROS) in cancer cells to a significantly higher extent and has an approximately five times effect on apoptosis [88]. Due to its anti-inflammatory effect, D-allose can also mitigate cisplatin-induced nephrotoxicity [89]. Moreover, D-allose acts as an immunosuppressant [90], inhibits ROS production from neutrophils [91] and has an inhibitory effect on ischemic lesions of the inner retina [92]. D-allose has a protective effect against liver ischemia reperfusion injury [93,94] and can be used to prevent osteoporosis by inhibiting osteoclast differentiation [95]. Furthermore, beneficial effects of D-allose have been reported against stroke [96], hypertension and obesity [78]. The safety of D-allose has also been demonstrated in preclinical trials in rats [97]. The authors indicated that D-allulose did not exhibit any toxicity. Apart from the low amounts that remained in the small intestine and cecum, D-allose was quickly absorbed from the digestive tract into the blood stream and expelled mainly through the urine [98].

D-tagatose is rare ketohexose, a C-4 epimer of fructose, which occurs naturally in the gum exudate of the cacao tree (*Sterculia setigera*), *Rocella* spp. [99,100] and in many foods, such as apples, oranges, and milk [101]. Currently, D-tagatose is produced on the industrial scale using a variety of methods which are constantly evolving. One of the most important means of its production involves Izumori’s enzymatic isomerization or isomerization of galactose under alkaline conditions, known as the Lobry de Bruyn–Alberda–van Ekenstein transformation [102,103]. D-tagatose was originally patented as a low-calorie sweetener and bulking agent [104]. D-tagatose is 8% less sweet than sucrose but contains only 1.5 kcal/g [105]. D-tagatose shows good properties in blends with other sweeteners, reducing mouth dryness and improving mouth-feel effects such as the reduction of sweet and bitter aftertastes [106]. It received GRAS approval in the USA in 2001 and in the EU in 2005 [107,108], and since then it has been used in various food ingredients, beverages, confectionery and dietary products [109]. D-tagatose has antioxidant and cryoprotectant properties [110], reduces body weight [111,112], and has a positive impact on dental health through its ability to inhibit biofilm formation and the coaggregation of the Streptococci and Actinomycetes involved in dental plaque formation [113]. D-tagatose has also therapeutic potential in type 2 diabetes [114], since it can improve glycemic control [115], but also acts as an antihyperglycemic agent [116].

Although the applications of rare sugars in human nutrition [101,109,117,118,119,120] and medicine [36,41,78,110] have been widely studied, there are an increasing number of reports highlighting their potential use for sustainable food production [1,121,122,123,124,125,126,127,128], suggesting a promising future for a potential application of rare sugars in agriculture.

## 3. Role of Rare Sugars in Plants

Using the plant model Arabidopsis, it has been shown that certain rare sugars can exhibit a herbicidal effect [129]. However, they can also inhibit the growth of some plant species such as mung bean, mustard, fenugreek, wheat [130], lettuce [131], rice [121], cress, Italian ryegrass [132], and many other species [133]. Another trait of rare sugars is the stimulation of defense-related genes of plants, for instance in citrus [134], Arabidopsis [135], rice [121], and tomato [125]. Rare sugars appear to have a dual activity on plants (growth inhibition and induction of disease resistance) [136]. The combination of these two effects could be very useful in praxis, in the case that rare sugars prove to be a potential source of growth retardant [122]. This opens the doors wide to the application of rare sugars in crop protection as new generation herbicides, plant growth regulators and resistance inducers for a more sustainable agriculture. However, further studies are still needed to understand the mechanisms underlying growth inhibition and resistance induction [122].

### 3.1. Rare Sugars as Herbicides and Plant Growth Regulators

Plant growth regulator residues can display toxic effects on human health [137] and their negative effects have been associated to liver and kidney problems, genetic mutations, damage to the nervous system and embryos, carcinogenic effects and impacts on reproductive potential [138]. Likewise, synthetic herbicides used for weed control could have negative impacts on the environment due to biodiversity reduction and accumulation in soil and water [139]. However, no herbicides with novel mode of action have been identified in the past 30 years [140] and the number of weeds showing resistance to herbicide molecules is growing [141,142]. Therefore, alternative solutions for weed control are required in order to reduce impacts on ecosystem and soil health [143], and rare sugars could contribute to solving these issues [129]. For instance, the rare monosaccharide 7-deoxy-sedoheptulose showed a strong herbicidal activity against Arabidopsis [129]. This herbicidal activity is highly effective even when applied at low dose rates (260 µM), without any cytotoxic impact on mammalian cells [129]. The 7-deoxy-sedoheptulose activity is based on the inhibition of the 3-dehydroquinate synthase involved in the shikimate pathway [129]. Unlike other known inhibitors of 3-dehydroquinate synthase (e.g., D-gluco-heptulosonate 7-phosphonate, 3-deoxy-D-arabino-heptulosonate 7-phosphonate, carbaphosphonate and its cyclohexenyl derivatives), 7-deoxy-sedoheptulose has shown its herbicidelike properties in vivo, and a great potential for its production in simple and scalable way [129]. Nevertheless, additional studies regarding the mechanisms of action on plants of agronomic interest, ecotoxic properties and economic sustainability of the 7-deoxy-sedoheptulose remain to be carried out.

**Figure 3:**
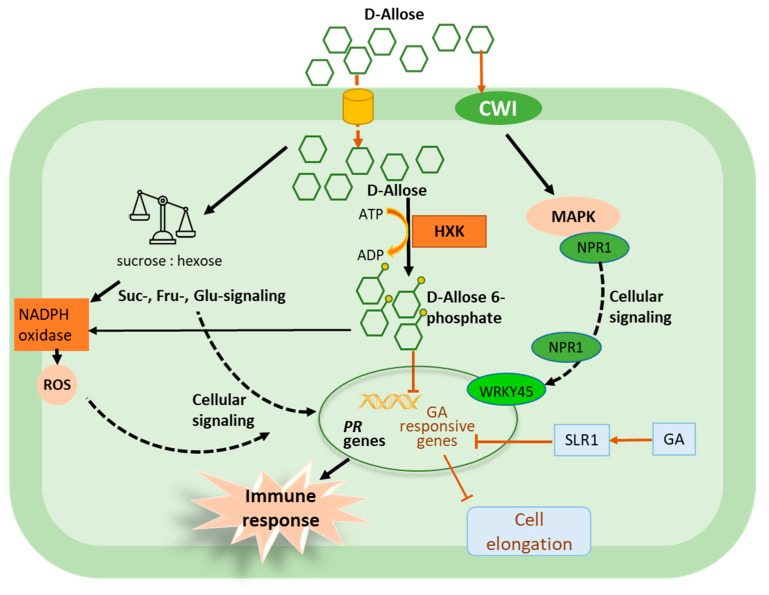
Schematic presentation of D-allose-triggered plant immunity and growth inhibition. After treatment D-allose can enter the plant cells through unknown mechanism, then phosphorylated by hexokinase (HXK) to produce D-allose 6-phosphate, which acts on the signal transduction downstream of DELLA protein (SLR1 in rice) as a suppressor of growth and gibberellin-dependent reactions. D-Allose can also activate NADPH oxidase leading to ROS production and resistance against *Xanthomonas oryzae* pv. oryzae [124]. Exogenous application of D-allose could also transiently upregulate extracellular cell wall invertase (CWI) activity, as reported for some nonmetabolizable sugars [152], resulting in activation of MAPK signaling pathway, transcription factors and cofactors such as WRKY 45 or Nonexpresser of PR (NPR1) genes—a key regulator of salicylic acid-mediated systemic acquired resistance pathway, and ultimately production of PR proteins. Penetration of D-allose inside the plant cell would also alter the sucrose:hexose ratio, thereby affecting the sugar signaling and triggering plant immune response.

Increasing evidence has also shown that D-allulose is able to inhibit the seed germination and growth of various plant species including mung bean, mustard, fenugreek and wheat [130]. The inhibitory effect of D-allulose on the growth of lettuce roots was shown to be dose-dependent (from 0.1 to 30 mM). This effect was abolished in the presence of sucrose in the growth medium [131]. Such results are in agreement with previous findings on Arabidopsis showing that mannose-induced growth inhibition can be abolished by adding metabolizable sugars [144,145]. The growth inhibitory effect of D-allose was also observed in rice shoots in a concentration-dependent manner, and the strongest effect (about 40% reduction) was found at the highest dose (1 mM) [121]. This effect was neither related to osmotic damage [131] nor caused by the rare sugars D-altrose and D-sorbose [121]. However, the possible mechanism of action of D-allose could rely on the induction of plant defense responses [146], a process known to allow decreases in plant fitness. For example, stunted growth is commonly observed in rice mutants that express constitutive defense responses or plants overexpressing defense-related genes [147,148,149,150,151]. It has also been shown that transcription factors that are simultaneously involved in the regulation of plant growth and defense response are upregulated by D-allose in rice (Figure 3) [121]. Moreover, D-allose, instead of inducing gibberellic acid production, suppressed the expression of gibberellin-responsive genes located downstream of the DELLA protein Slender Rice1 (SLR1) through the HXK-dependent pathway [146], and modulated the expression of the abscisic acid signaling genes [146], indicating the complex impacts of rare sugars on phytohormone-related signaling pathways.

Although there is a potential growth retardant activity of rare sugars, the factors limiting their further development in practice might result from the required high dose rates [133]. For example, D-allose had to be applied at dose rates higher than 3 and 10 mM to inhibit the growth of lettuce roots and hypocotyls, respectively [153]. The inhibition of plant growth by D-allose was shown to be enhanced in the presence of nonionic and biodegradable unbranched alkyl chains, such as sugar fatty acid esters [153]. Compared to pure D-allose, D-allose fatty acid esters have stronger biological activity because their carbon chains are hydrophobic, and therefore improve surface activity and membrane permeability [153,154,155]. The efficacy of D-allose fatty acid esters was further improved by prolonging the carbon chain of the fatty acid moiety, which significantly enhanced the inhibitory activity of 6-O-dodecanoyl-D-allose regarding the rice growth compared to octanoate and decanoate [156]. Moreover, it has been shown that the α-axial hydroxyl group at the C-3 location of D-allose ester played an important role in the plant growth inhibitory effect, since the β-hydroxy group at C-2 or C-4 did not significantly affect the inhibitory activity of D-allose ester [157]. It has, however, been shown that all ester groups (dodecanoates, octanoates and decanoates), used in Kobayashi’s study [158] to improve the growth inhibition activity of D-allose, are sensitive to hydrolysis by esterases [159]. Other studies [158,159] highlighted the importance of the amide group instead of esters for the efficacy of D-allose derivatives. For example, the amide 6-decanoylamino-6-deoxy-D-allose had a weaker growth inhibitory capacity compared to 6-O-Decyl-D-allose [159]. Therefore, 6-(decanoylamino)-1,2,6-trideoxy-D-allose is very effective in inhibiting the growth of lettuce, cress, Italian ryegrass, and rice seedlings in a dose-dependent manner and with significantly higher efficacy compared to its corresponding deoxy-D-allose ester (6-O-decanoyl-1,2-dideoxy-D-allose) [158]. Furthermore, when applied with gibberellic acid, 6-decanoylamino-1,2,6-trideoxy-D-allose exerted a significant inhibitory effect on gibberellic acid biosynthesis [158].

### 3.2. Rare Sugars as Plant Resistance Inducers

Over the last few decades, the implementation of biobased elicitors of plant resistance has constituted an innovative ecofriendly strategy for biocontrol of plant diseases. Increasing interest has been devoted to sugar-based molecules for their roles in plant immunity through their interaction with plant metabolism, sugar transport and as signaling molecules [160,161,162,163]. Plants are able to recognize PAMPs (pathogen-associated molecular patterns) or pathogen effectors, which lead to the activation of PAMP-triggered immunity (PTI) and effector-triggered immunity (ETI) [164], resulting in most cases in induced plant resistance against various pathogens. Most of the sugar-based molecules are oligosaccharides derived from the plant cell wall, or from beneficial or pathogenic microbes. They have been used for crop farming as biopesticides, biofertilizers, for seed coating formulation, and agricultural film [165,166,167]. The frequently investigated sugar-based molecules include α-1,4-oligogalacturonides [168,169,170,171], chitooligosaccharides [172,173,174,175], β-1,3-glucans [176,177,178,179], xyloglucans [180], lipopolysaccharides [181,182,183], and rhamnolipids [184]. Simple sugars have also been shown to be efficient resistance inducers, such as turanose and fluorosucrose [185], sucrose [186,187], galactinol and raffinose [188]. These plant resistance inducers can either elicit a broad range of defense responses, including the upregulation of defense genes, the production of ROS, the activation of MAPKs and the production of phytoalexins, or prime plants for enhanced faster and stronger responses after subsequent pathogen challenge [189,190,191,192].

Some rare sugars have also been identified as effective inducers of plant immune response and resistance against various pathogens. D-allulose induced the upregulation of defense-related genes and resistance in rice against bacterial blight caused by *Xanthomonas oryzae* pv. oryzae [122,134]. D-allose also induced the expression of PR-1 and PDF1.2 genes in Arabidopsis [135] and enhanced the resistance of rice against bacterial blight [121]. The efficacy of D-allose to reduce bacterial blight disease in rice was high (approximately 70–80%) compared to D-glucose, D-fructose, D-altrose and D-sorbose [121]. The effect of D-allose is achieved through the stimulation of plant defense mechanisms, including the upregulation of hundred defense-related genes, such as those encoding probenazole-inducible protein, pathogenesis related protein 1 (PR-1), proteinase inhibitor, lipoxygenase, peroxidase, β-1,3-glucanase and chitinase. It has also been reported that D-allulose induced rice resistance and the upregulation of defensive genes in a dose-dependent manner [122]. However, the disease reduction conferred by D-allulose was lower compared to D-allose, and the amount of D-allulose needed to confer the same level of resistance was five times higher than that of D-allose [122]. Rice plants treated simultaneously with D-allose and ascorbic acid (ROS scavenger) did not exhibit any significant protection, suggesting a determinant role of ROS production in D-allose-induced resistance to bacterial blight in rice [123]. This is consistent with the observation that D-allose induces the *OsrbohC* gene, which encodes NADPH oxidase and belongs to the Respiratory burst oxidase homolog (Rboh) gene family involved in ROS production during plant–microbe interactions [193,194,195]. It has also been shown that rice plants overexpressing the *OsrbohC* gene were highly sensitive to D-allose treatment and expressed weaker disease symptoms compared to the wild type [123], suggesting that D-allose induces rice resistance to *X. oryzae* pv. oryzae by activating NADPH oxidase (Figure 3). In addition, phosphorylation of D-allose at the C-6 level seemed to be crucial for activating plant resistance, since treatments with a hexokinase inhibitor (N-acetylglucosamine) reduced the efficacy of D-allose-induced resistance [123]. Likewise, the D-allose derivative called 6-Deoxy-d-allose, which cannot be phosphorylated, failed to induce resistance in rice [123], suggesting that phosphorylation is a crucial step for the functional activity of rare sugars (Figure 3). D-allose induced tomato resistance against grey mold and bacterial speck through the increased production of ROS and the priming-enhanced expression of PR genes (e.g., PR1a, PR2a and PR3b) after subsequent inoculation with *Botrytis cinerea* and *Pseudomonas syringae* pv. tomato [125]. Treatment with such a resistance inducer, primed plant cells and it did not directly induce defense-related genes before infection. Therefore, plants may have few energetic trade-offs as defense signaling was not upregulated [196] after rare sugar treatment. A recent study [128] also examined the impact of D-tagatose on the immune system of cucumber, rice and Arabidopsis. The expression patterns of the investigated defense-related genes did not show any typical induction/or reduction following the treatment. The authors concluded that D-tagatose apparently does not impact plant immunity as a defense activator. However, owning to its strong protective effect against various diseases and its obvious direct effect on pathogens, it is hypothesized that treated plants could have an alternative defense strategy against pathogens, which can be weakened by D-tagatose [128].

Rare sugars could also induce plant disease resistance possibly by interfering with cell wall invertases (CWI) and the hexose:sucrose ratio [185]. The induction of CWI activity is essential to balance the sugar partitioning between plant defense reactions and pathogens development [197,198]. The loss of function of the CWI gene in rice results in a loss of resistance to postharvest pathogens [199]. However, the constitutive expression of CWI enhances the resistance to pathogens by activating the plant defense responses, including enhanced expression of PR genes and transcription factors in rice [199], tobacco [200,201,202] and tomato [203]. It has been reported that the naturally occurring sugar analogue 2,5-dihydroxymethyl-3,4-hydroxypyrrolidine (DMDP) can also stimulate the plant immune system [185]. DMDP also inhibits invertase activity [204] and alters the hexose:sucrose ratio [205]. Thus, DMDP would impact the glucose [206], fructose [207], and sucrose-specific signaling pathways [208], thereby modulating plant defense responses [185]. D-allulose [130,209] was also able to penetrate the cell and might potentially affect the plant’s defense responses in a similar manner to DMDP, by altering the hexose:sucrose ratio, or even interfering with CWI activity. Nevertheless, the mode of action of D-allose remains to be elucidated, especially to clarify its role in possible interaction with the sucrose:hexose ratio and activation of the plant immune system [9]. It has also been shown that D-allulose is phosphorylated by hexokinase and fructokinase to D-allose-6-phosphate in lettuce [131], without further metabolization. Therefore, as postulated in the concept of sweet immunity, rare sugars might have a similar effect on plants as nonmetabolizable sugars such as sucrose isomers palatinose and turanose, and fluoro-sucrose (a sucrose analogue) [185]. The latter activated MAPKs and transiently induced the expression of extracellular invertases in tomato cell culture, resembling the fungal elicitor effect [152].

## 4. Rare Sugars as Sustainable Control Agents against Crop Pests and Diseases

Rare sugars can inhibit plant pathogens with different lifestyles [128,210,211]. This effect has been observed in various pathosystems including downy mildews in onion, spinach, cabbage, Chinese cabbage, cucumber and grapevine [128,210], powdery mildews in cucumber, barley, pepper, tomato, eggplant, apple, grapevine [210,211] and strawberry [128], grey mold in tomato [210], Alternaria sooty spot in cabbage and brown spot in rice [128], cucumber anthracnose [128], rice blast and brown rust of wheat [210], and sheath blight in rice [128]. Rare sugars have shown a biocidal activity against ants and houseflies, but also against important crop pests such as bruchid beetle (*Callosobruchus maculatus*) [6,212], desert and migratory locust (*Schistocerca gregatoria* and *Locusta migratoria*), moths of Spodoptera spp., *Heliothis virescens*, and *Helicoverpa armigera* [213,214], making them interesting candidates for new generation of sustainable nematicides, insecticides, fungicides, and bactericides for more sustainable agriculture.

### 4.1. Rare Sugars as Sustainable Fungicides

**Figure 4:**
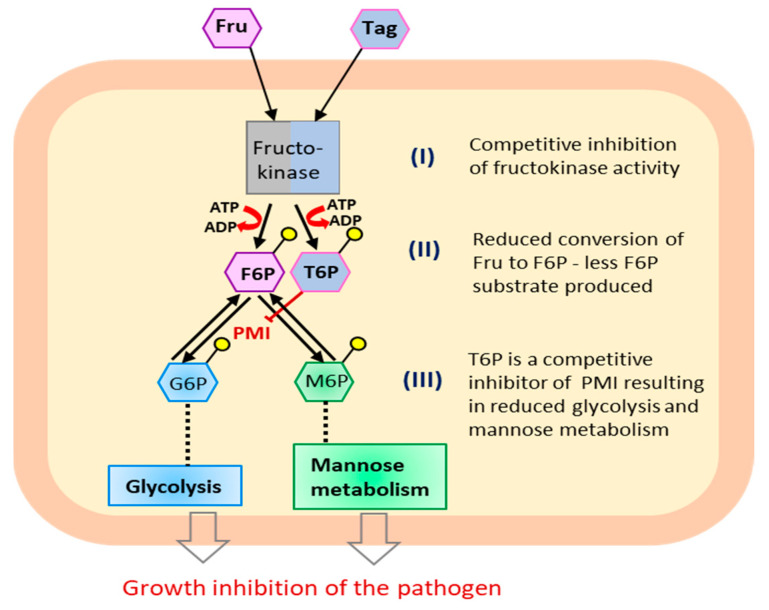
Proposed mechanism involved in fungicide effect of D-tagatose (Tag) according to Mochizuki et al. [128]. Three steps of Tag chain-inhibitory effects on multiple targets in *Hyaloperonospora arabidopsidis*, causal agent of Arabidopsis downy mildew: (**I**) through competitive inhibition of fructokinase activity, Tag inhibits the first step of mannose metabolism-the phosphorylation of D-fructose (Fru) to D-fructose 6-phosphate (F6P) by fructokinase; (**II**) conversion of Tag to D-tagatose 6-phosphate (T6P) will reduce the conversion of Fru to F6P-less F6P means less substrate for glycolisis and mannose metabolism; (**III**) The produced T6P acts as a competitive inhibitor of phosphomannose isomerase (PMI) reducing glycolysis and mannose metabolism. Through steps I-III Tag inhibits the pathogen growth and disease development.

D-tagatose is a well-known rare sugar with antifungal and antioomycete activities [128,210]. D-tagatose had inhibitory activity against some important pathogens, such as tomato late blight, grapevine downy mildew, rice blast and seedling blight, cucumber damping-off and powdery mildew [210]. However, the study regarding the mode of action of D-tagatose is poorly understood and further studies are needed to understand the mechanisms underlying pathogen growth inhibition. Additionally, D-tagatose did not affect the mycelial growth of *Aspergillus niger*, *Cladosporium cladosporioides*, and *Penicillium chrysogenum* [215], while it promoted the spore germination of *A. niger* [216], thus displaying a selective effect on the growth of plant-associated microorganisms. Moreover, the growth of *Trichoderma harzianum* and *T. pleuroticola* was strengthened in the presence of D-tagatose, but not the growth of *T. pleurotum* [217], meaning that D-tagatose has nutritional or antinutritional effects on microorganisms within species that belong to the same genus. It has also shown that D-tagatose reduces the severity of a wide variety of economically important crop diseases in both pot and field trials, such as downy and powdery mildews in grapevine, cucumber, Chinese cabbage, onion, and spinach at concentrations ranging from 0.5% to 1% [128]. The efficacy of D-tagatose against cucumber downy mildew was comparable to that of chemical fungicides [128], by acting directly on pathogens, rather than activating plant defense mechanisms [128] (Figure 4).

D-tagatose inhibited the hyphal growth of *H. arabidopsidis* [128], which is linked to a competitive inhibition of fructokinase, the first enzyme of sugar metabolism that phosphorylates the C-6 of D-fructose and D-tagatose. After phosphorylation of D-tagatose by fructokinase, D-tagatose 6-phosphate will also act as a competitive inhibitor of phosphomannose isomerase that produces D-glucose 6-phosphate and D-mannose 6-phosphate [128]. In such way, D-tagatose interferes with the metabolic pathways of *H. arabidopsidis* at multiple target sites (glycolysis and mannan/mannoglucan synthesis). Moreover, D-tagatose caused severe ultrastructural alterations of *Phytophthora infestans*, such as the formation of circular and concentric mitochondrial cristae, and inhibited hyphal growth with a decreased ATP content and oxygen consumption rate [127]. At the same time, ROS accumulation and the expression of apoptosis and oxidative stress-related genes were increased by D-tagatose in *P. infestans*, but not in *P. cinnamomi* [127], corroborating the species-specific antinutritional effects of D-tagatose [217].

### 4.2. Rare Sugars as Sustainable Nematicides

Plant-parasitic nematodes are widely considered one of the a major threats to the agriculture production, since they cause severe crop losses worldwide [218]. Since the use of methyl bromide, organophosphate and carbamate as soil fumigants was banned, sustainable methods of nematode control have been gaining popularity [219]. D-allulose was shown to be able to inhibit the motility, growth and egg bearing rate of *Caenorhabditis elegans*, probably by interfering with nematode nutrition [66]. It has also been shown that D-arabinose, D-allose, D-talose and L-idose inhibited *C. elegans* growth under the monoxenic and axenic conditions [1,2]. Likewise, 1-Deoxy-D-allulose can drastically reduce the growth of *C. elegans* [220]. Furthermore, it has been reported that 5-deoxy-and 6-deoxy-D-allulose could not inhibit the growth of *C. elegans*, indicating that the growth inhibiting effects are dependent on the sugar stereoisomeric structure [220]. Such studies open new doors to the use of rare sugars as sustainable nematicides. The authors also showed that the D-arabinose-induced growth inhibition was reversed when D-ribose or D-fructose, but not D-glucose, were added to the medium [1,220], suggesting that cross interactions between rare sugars and the common sugar metabolism could be responsible for the nematode growth inhibition.

**Figure 5:**
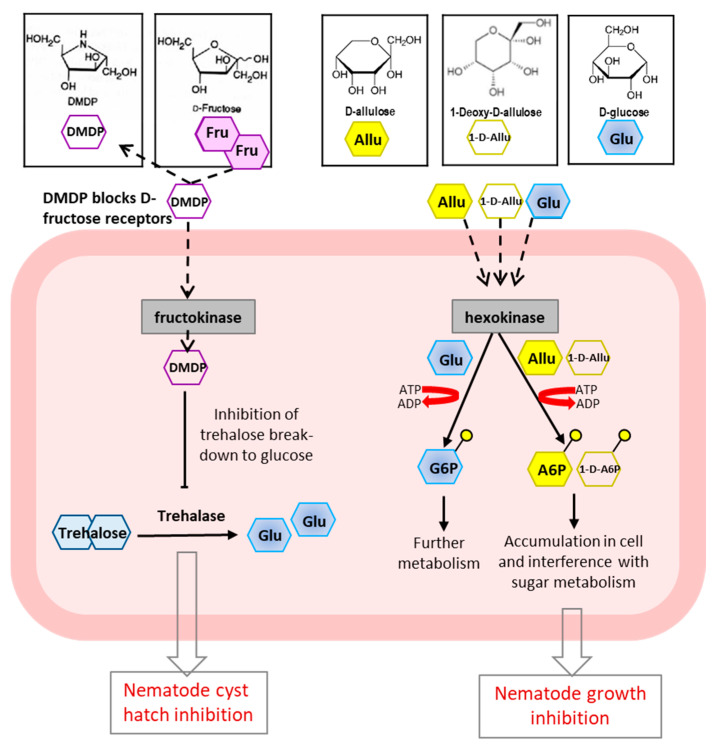
Possible modes of action of fructose analogue-sugar-like 2,5-dideoxy-2,5-imino-D-mannitol (DMDP) and D-allulose/1-Deoxy-D-allulose on nematodes. DMDP (left side), due to its high structural similarity with fructose temporarily blocks the fructose receptors like fruktokinases and in such way disturbs the fructose sensing and signaling pathways. DMDP can be taken up inside the cells where it interferes with the trehalose breakdown and inhibits cyst hatch of nematodes (e.g., potato cyst nematode). On the right side of the figure is the possible *C. elegans* growth inhibitory action of D-allulose and 1-Deoxy-D-allulose. D-Glc taken up by cells is phosphorylated by hexokinase and D-Glc-6-phosphate is formed through glycolysis. D-Allulose and its deoxy derivate are also phosphorylated by hexokinase; however, the cell is not able to entirely metabolize the newly formed D-allulose/1-Deoxy-D-allulose-6-phosphate. Instead, they accumulate in the cell, inhibiting glycolytic enzymes, thus interfering with sugar metabolism [2].

Due to their structural similarity, D-arabinose could be integrated into nucleotides or cofactors instead of D-ribose [1]. This would lead not only to deficiency of essential functional metabolites, but also to the production of non-natural metabolites with an antimetabolite role [1,221]. Given that D-fructose treatment, like D-ribose treatment, abolished the D-arabinose-induced growth inhibition, this suggests that, due to their structural similarity, D-arabinose interferes with D-fructose metabolism [1]. Another possibility would be that cells use D-fructose in order to synthetize the depleted D-ribose [220]. The authors reported that 1-deoxy-D-allulose (1d-D-allulose) was effective in inhibiting *C. elegans* growth at low concentrations, while the combination of 1d-D-allulose and D-fructose overturned the inhibitory effect [220]. Growth inhibition was abolished possibly because D-fructose substituted the depleted D-ribose, since D-fructose is preferentially metabolized by the cells for D-ribose synthesis [222]. In the case of D-allulose/1-deoxy-D-allulose-induced growth inhibition of *C. elegans*, it is assumed that, following phosphorylation, D-allulose/1-Deoxy-D-allulose-6-phosphate accumulates in the cells instead of being metabolized, impeding glycolysis reactions [2]. Since these rare sugars share the structural similarity with natural substrate D-glucose (for D-allose and L-idose), D-galactose and D-manose (for D-talose), it can be speculated that the growth-inhibitory effect could rely on the antinutritional function in carbohydrate metabolism [2]. A parallel can be drawn from the medical studies, where it has been noted that D-allose has an effect on the proliferation of cancer cells [81,88] by upregulating the expression of thioredoxin-interacting protein (TXNIP). Its mode of action is based on decreasing glucose uptake by carcinoma cells until they stop growing [223]. It has been found that the D-allose-mediated induction of TXNIP will promote the downregulation of the glucose transporter 1 (GLUT1), which is responsible for glucose uptake, preventing glucose absorption by cancer cells [223]. These results suggest the antimetabolite function of D-allose in the interference with sugar metabolism and with the signaling pathways of energy metabolism through the TXNIP [2]. Another example is the antinematode activity of 2,5-dideoxy-2,5-imino-D-mannitol, which inhibits the hatch cysts, caused by *Globodera pallida*, and immobilizes the juvenile stage of *Globodera rostochiensi* [224]. Drenching the roots with 2,5-dideoxy-2,5-imino-D-mannitol was the most effective application strategy against the root galling of tomato caused *Meloidogyne javanica* [224]. Moreover, treatments with 2,5-dideoxy-2,5-imino-D-mannitol limited the ability of *Xiphinema diversicaudatum* to acquire Arabis Mosaic Virus and to transmit it to petunia seedlings [224]. Although it should be further validated, the dose-independent activity of 2,5-dideoxy-2,5-imino-D-mannitol suggests that its mode of action might involve the activation of possible receptors in nematode cells [224]. Moreover, it was reported that 2,5-dideoxy-2,5-imino-D-mannitol can inhibit trehalose breakdown by interfering with trehalase [225] (Figure 5).

### 4.3. Rare Sugars as Sustainable Insecticides

Insects and other arthropods are estimated to be responsible for a loss of approximately 5 to 20% of the annual crop production worldwide [226,227]. Although highly effective, synthetic insecticides can be harmful to human health and have a strong tendency to accumulate in the environment and disturb ecosystems due to their high toxicity on nontarget organisms [228]. Therefore, novel alternative methods are needed and some carbohydrates (e.g., sucrose) have shown potential activities against insects, such as *Cydia pomonella* [229]. The mode of action of sucrose was shown to be linked to the induction of resistance by antixenosis to egg laying insects [230]. Based on the observation on common sugars, it is not surprising that rare sugars may also be great candidates for the next-generation of sustainable insecticides. It has been shown that DMDP inhibits the gut alpha-glucosidase enzymes of insects and is toxic to the larvae of the bruchid beetle *Callosobruchus maculatus* at very low concentrations [212]. It was demonstrated that DMDP can be harmful when it is ingested by *Spodoptera littoralis* [213]. On the other hand, a high dose of DMDP (1 mg/L g body weight) had no effect on *Schistocerca gregatoria* and *Locusta migratoria* [213]. It has also been shown that the gut of *S. gregaria* can act as a barrier to the influx of dietary tannins into the hemolymph; therefore, it can be suspected to have a similar mechanism working against DMDP [213]. DMDP has been shown to be a potential insect deterrent [225] and caused a reduced response to phagostimuli of glucose, sucrose and fructose in several insect species, such as *S. gregatoria*, *L. migratoria*, *S. littoralis*, *Spodoptera frugiperda*, *Heliothis virescens*, and *Helicoverpa armigera* [213,214]. Thus, the mode of action underlying the effect of DMDP may be related to the similar size and shape of DMDP compared to fructose, leading to the scenario where DMDP temporarily blocks the fructose receptor sites [224]. Levin and Zehner [231] showed that the rare sugar L-fructose is an effective biocide for ants and houseflies, and that D-sorbose would have anti-insect effect [6]. Like the phyllosphere microbiota [167], the insects would not remain passive following treatments with rare sugars in the field. It is possible that rare sugars have nutritional or antinutritional effects on different insect species, as shown for microbial populations [126]. In such a way, rare sugars might be used as initial sources of nutrients, boosting or suppressing the insect population build-up. It is expected that rare sugars could attract certain (possibly beneficial) insect species, and thus provide additional ecological properties, but further studies are required on these aspects.

## 5. Conclusions

Sugars are involved in various metabolic and signaling pathways, including those that contribute to plant defense against pests and pathogens. The exogenous application of rare sugars appears to be a valid strategy for stimulating plant immunity or for inhibiting phytopathogens. However, possible limitations of using rare sugars in practical crop protection can be predicted, such as low penetration through the cuticular barrier due to polar properties, low rainfastness due to high solubility, and high doses needed for their sufficient efficacy [132,167]. Rare sugars would encounter the same problems that can probably be solved by using the right formulations. Ohara’s patent [232] offered a list of various coformulants that improve the efficacy of rare sugars such as D-tagatose, D-allose, D-allulose, D-talose, D-sorbose, D-galactose, L-fructose, D-mannose and D-mannitol. Recently, a drastic improvement in the efficacy of D-tagatose due to its formulation was reported [128], suggesting the importance of further functional and molecular mechanisms triggered by formulated rare sugars in plants. The prebiotic properties of D-tagatose on leaf-associated microbial communities [126] and the possible nutritional effect of rare sugars on some microbial taxa [215,216,217] suggested a possible degradation of rare sugars by natural microorganisms.

Although rare sugars are largely used in human and are well-known beneficial molecules for human health, recent evidence also suggests their potential application for a more sustainable agriculture. In particular, some rare sugars were able to regulate plant growth, stimulate crop resistance, inhibit plant pathogens, control insects and nematodes with large potential for applications in crop management and protection. However, deep studies on the mode of action, stability under field conditions, possible degradation by indigenous microorganisms and environmental fate of rare sugars are required to further develop their application in practice.
